# Time-related factors predicting a positive response to cardiac resynchronisation therapy in patients with heart failure

**DOI:** 10.1038/s41598-023-35174-9

**Published:** 2023-05-26

**Authors:** Jacek Wilczek, Tomasz Jadczyk, Wojciech Wojakowski, Krzysztof S. Gołba

**Affiliations:** 1 Department of Electrocardiology and Heart Failure, Medical University of Silesia, Katowice, Poland; 2 Electrocardiology Department, Upper Silesian Medical Center, Katowice, Poland; 3 Division of Cardiology and Structural Heart Diseases, Medical University of Silesia, Katowice, Poland; 4 Third Department of Cardiology, Upper Silesian Medical Center, Katowice, Poland; 5 Interventional Cardiac Electrophysiology Group, International Clinical Research Center, St. Anne’s University Hospital in Brno, Brno, Czech Republic

**Keywords:** Cardiology, Diseases, Pathogenesis, Risk factors

## Abstract

This study aimed to identify time parameters predicting favourable CRT response. A total of 38 patients with ischemic cardiomyopathy, qualified for CRT implantation, were enrolled in the study. A 15% reduction in indexed end-systolic volume after 6 months was a criterion for a positive response to CRT. We evaluated QRS duration, measured from a standard ECG before and after CRT implantation and obtained from mapping with NOGA XP system (AEMM); and the delay, measured with the implanted device algorithm (DCD) and its change after 6 months (ΔDCD); and selected delay parameters between the left and right ventricles based on AEMM data. A total of 24 patients presented with a positive response to CRT versus 9 non-responders. After CRT implantation, we observed differences between responders and non-responders group in the reduction of QRS duration (31 ms vs. 16 ms), duration of paced QRS (123 ms vs. 142 ms), and the change of ΔDCDMaximum (4.9 ms vs. 0.44 ms) and ΔDCDMean (7.7 ms vs. 0.9 ms). The difference in selected parameters obtained during AEMM in both groups was related to interventricular delay (40.3 ms vs. 18.6 ms). Concerning local activation time and left ventricular activation time, we analysed the delays in individual left ventricular segments. Predominant activation delay of the posterior wall middle segment was associated with a better response to CRT. Some AEMM parameters, paced QRS time of less than 120 ms and reduction of QRS duration greater than 20 ms predict the response to CRT. ΔDCD is associated with favourable electrical and structural remodelling.

***Clinical trial registration*:** SUM No. KNW/0022/KB1/17/15.

## Introduction

Heart failure has one of the highest morbidity and mortality rates globally. It is estimated that approximately 2% of the population suffers from symptomatic heart failure, and this percentage increases with age^1^. Conduction disturbances underlie the dyssynchrony of cardiac contraction and cause haemodynamic consequences that lead to unfavourable remodeling^2^. Cardiac resynchronization therapy (CRT) restores the desired sequence of heart contraction, improves quality of life, reduces severity of symptoms, and decreases morbidity and mortality, but in approximately 30% of patients it fails to produce the expected hemodynamic and clinical effects^3^. The reasons for the lack of response to CRT are still being investigated, and efforts are being made to identify predictive parameters to identify those patients who may benefit from biventricular pacing. The present study attempts to evaluate parameters that determine the distribution of electrical stimulation in the heart before and after implantation of a cardiac resynchronization system. The study also evaluates changes in some of them 6 months after implantation. Thus, this study aimed to identify the time parameters that may predict the response to CRT.

## Material and methods

Between April 2014 and July 2017, 38 patient—9 women (24%) and 29 men (76%), aged 49 to 76 years (mean age 65.6 ± SD 5.7)—with sinus rhythm, left bundle branch block (LBBB), ischemic cardiomyopathy, and with ESC class I and II indications for CRT implantation were included in the study. Participants with the following factors were excluded from the study: acute coronary syndrome less than 3 months prior to study inclusion, coronary artery disease requiring revascularization, previously implanted pacemaker or cardioverter-defibrillator, aortic valve calcification or left ventricle (LV) thrombus, chronic kidney disease, pregnancy or lactation, active neoplastic disease, viral infection, hemorrhagic diathesis, allergy to contrast solution, current participation in another study, or life expectancy less than 6 months. All methods were performed in accordance with the relevant guidelines and regulations. The investigation conforms to the principles outlined in the Declaration of Helsinki. The project was approved by the Bioethics Committee of the Medical University of Silesia and registered under number KNW/0022/KB1/17/15. All patients signed the informed consent form. All patients underwent echocardiography with Phillips iE 33 and Cx 50 devices. Anatomo-electromechanical mapping (AEMM) was performed in each patient using the NOGA XP system. Unipolar and bipolar left ventricular electrical potentials (273 ± 47 recorded and mapped points per each patient), as well as time and mechanical parameters, were recorded from each mapped point. Averaged parameter values were analysed in the LV “bull’s-eye” view in 4 basal and intermediate segments (anterior, septal, posterior, and lateral) and in 1 apical segment. The resynchronization system implantation procedure was performed in all patients with the right ventricular lead positioned in the RV apex and the left ventricular lead positioned in the target lateral or posterolateral cardiac vein, avoiding the apical region. The function of the resynchronization system was confirmed four weeks after the procedure. Echocardiography and clinical evaluation were repeated 6 months after the implantation. The excitation propagation delay parameters were analysed based on three independent measurements.

1. QRS complex measurements.
  1. The QRS duration (QRSd) and the paced QRS (QRSp) were measured from the standard ECG recording at 50 mm/s, based on the QRS duration in V1–V6 leads at baseline and after 6 months of pacing and the difference was calculated between the native QRS (QRSd) and the paced (QRSp) complexes—ΔQRS.
  2. The QRS complex time from the ECGs was obtained during NOGA electromechanical mapping (QRSn) in leads V1–V6 using individual optimal signal gain, which allowed precise detection of the QRS complex onset and termination within the isoelectric line.
2. The delay measured by the CRT Toolkit Auto VectSelekt Quartet™ MultiVector Test algorithm—Device Calculated Delay (DCD) between the left and right ventricles available on the Abbott Quadra Assura MP cardioverter-defibrillators used in this project is based on the measuring the beat detection delay between the right ventricular lead and individual rings of the quadripolar left ventricular lead (D1, M2, M3, P4). A test version of native beat delay detection was performed immediately after implantation and after 6 months with assessment of its change—ΔDCD.
3. Left and right ventricular delays were also measured using parameters obtained with the NOGA XP system (AEMM):
  1. Intraventricular delay (IVd) is defined as the delay between the earliest recorded QRS excursion in V1 and the earliest left ventricular excitation recorded by the catheter within the left ventricle (LV).
  2. Left ventricular electrical activation time (LVEAT) is defined as the time between the earliest and the latest recorded potential within the left ventricle.
  3. Total activation time (TAT) is defined as the sum of the interventricular delay time and the left ventricular electrical activation time, reflecting right and left ventricular activation.
  4. Local activation time (LAT)—mean and maximum LAT values—represents the time between the earliest activation within the left ventricle and the activation of the corresponding left ventricular segment.

### Statistical analysis

In the statistical analysis, the Shapiro–Wilk test was used to assess normality of distribution, the Student's t-test was used for independent samples, and the U-Mann Whitney test was used to compare the groups. Weighted univariate linear regression analysis was used to assess the relationship between selected parameters, and a model based on logistic regression analysis, C statistics and ROC analysis was used in the prediction evaluation.

Calculations were performed using MedCalc® statistical software version 20.106-32-bit.

## Results

**Figure 1:**
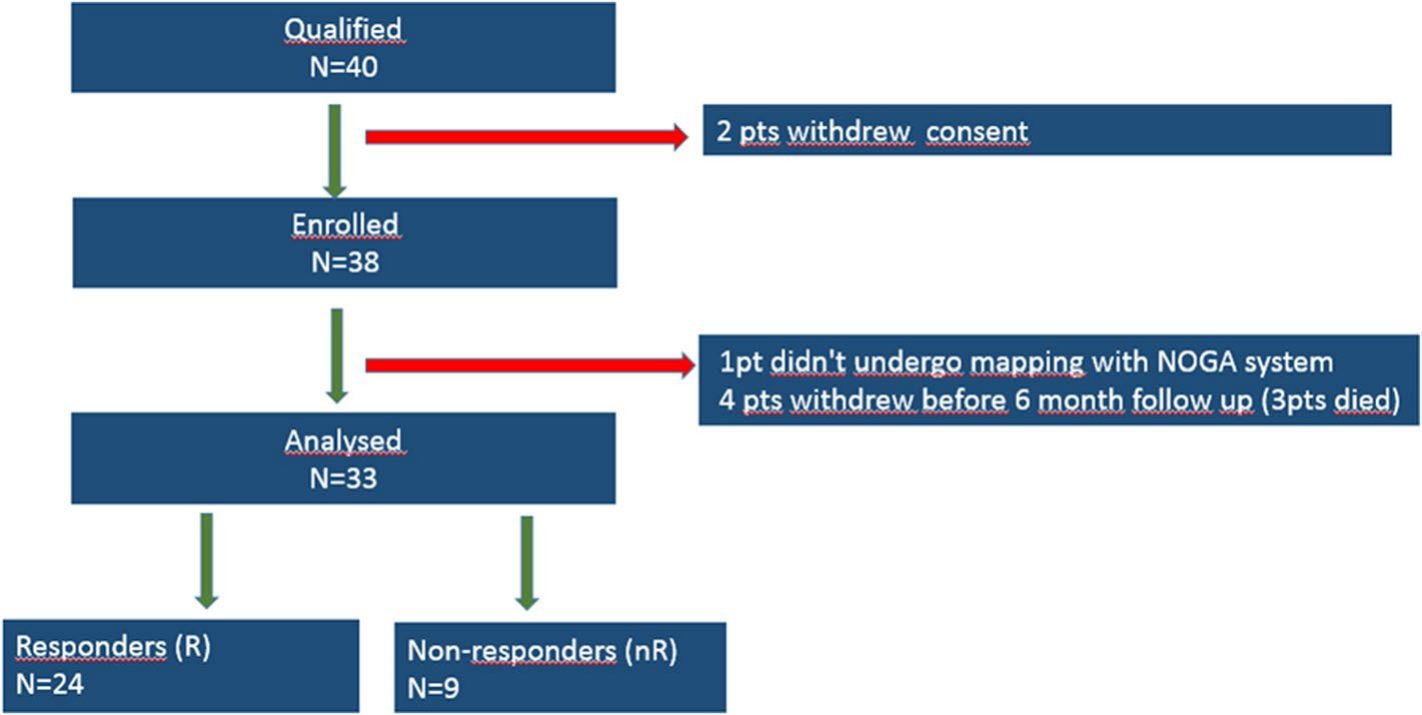
Study group.

Forty patients were eligible for the study, 38 were enrolled, and 33 of them were analyzed (Fig. 1).

The characteristics of the patient group is shown in Table 1.

**Table 1:** Characteristics of the study group.

Age	65.75 ± 5.95
Gender: Female/Male	7 (21%) / 26 (79%)
Weight	86.36 ± 15
BMI	30.23 ± 4.3
Etiology HF (ischemic)	33(100%)
Medications	
B-blocker	33 (100%)
ACEI/ARB	33 (100%)
Spirinolactone/eplerenone	31/33 (94%)
Loop diuretic	27/33 (82%)
Statine	32/33 (97%)
Comorbidities	
Previous myocardial infarction	30 (91%)
Hypertension	31 (94%)
Diabetes mellitus	16 (48%)
Chronic Obstructive Pulmonary Disease	2 (6%)
Atrial fibrillation	7 (2.94%)
% BiV pace at 6MFU	97 ± 3.7 (%)

ACEI, Angiotensin-converting enzyme inhibitors; ARB, Angiotensin receptor II blockers; %BiV pace, % biventricular pacing; BMI, Body mass index; 6MFU, six month follow-up.

After 6 months the changes in echocardiographic parameters were observed in relation to baseline values, including a reduction in mean indexed end-systolic volume (LVESVi) (92.47 ± SD21.1 vs. 71.88 ± SD29.7, *p* = 0.002). The criterion for a positive response to resynchronization therapy was a 15% improvement in LVESVi. Differences between responders (N = 24) and non-responders (N = 9) were also assessed in terms of the parameters studied and their relationship with change in LVESVi after 6 months of biventricular pacing (Table 2).

**Table 2:** Differences in analyzed parameters between responders (R) and non-responders (nR) and and their association with change in ESVI after 6 months of biventricular pacing.

Parameter (ms)	Differences in analyzed parameters between responders (R) and non-responders (nR) (mean ± SD )		*p*	Relationship of parameters with change in ESVI after 6 months biventricular pacing		
	Responders (R)	Non-responders (nR)		Regression equation	r	*p*
	N = 24	N = 9				
QRSd^1^ base	157.1 ± 10.8	157.8 ± 25.9	0.91	y = − 0.1 x + 39.5	0.10	0.59
QRS NOGA^1^	169.7 ± 11.7	172.6 ± 16.2	0.58	y = − 0.13x + 44.4	0.08	0.64
QRSp^1^	122.9 ± 17.3	142.2 ± 19.2	0.01	y = − 0.31x + 61.7	0.32	0.068
ΔQRSd^1^	31.2 ± 16.4	15.6 ± 13.3	0.005	y = 0.67x + 3.3	0.5	0.003
QRSp base_6M^2^	120.8 ± 14.7	142.2 ± 19.2	0.003	y = 0.75x-0.4	0.55	0.001
Total RV-LV activation time TAT^1^	149.0 ± 19.8	138.6 ± 24.5	0.21	y = 0.11x + 6.8	0.11	0.54
DCDmax base^1^	118.5 ± 27.4	112.0 ± 20.1	0.52	y = 0.2x − 0.6	0.21	0.24
DCDmean base^1^	109.4 ± 37.4	101.3 ± 19.3	0.43	y = 0.25x -3.5	0.25	0.16
ΔDCDMean base_6M^2^	7.7 ± 7.8	-0.9 ± 4.5	0.004	y = 1.92x + 13.1	0,60	< 0.001
IVD^1^	40.3 ± 26.7	12.6 ± 11.3	0.005	y = 0.44x + 8.3	0.49	0.003
ΔDCDMax base_6M^2^	4.9 ± 11.9	-0.4 ± 4.7	0.05	y = 1.16x + 19.7	0.51	0,004
LVEAT^1^	108.7 ± 19.4	113.7 ± 20.7	0.54	All		
				y = − 0.24x + 53.3	0.45	0.008
				R		
				y = − 0.38x + 73.2	0.49	0.016
				nR		
				y = 0.36x − 44.6	0,71	0.033
LATmax^1^	74.8 ± 18.8	76.2 ± 15.8	0.21	All		
				y = − 0.07x + 27.0	0.10	0.6
				R		
				y = − 0.32 x + 53.3	0.35	0.098
				nR		
				y = 0.88 x − 68.3	0.79	0.012
LATmean^1^	33.1 ± 9.6	39.4 ± 7.3	0.08	All		
				y = -0.37 x + 35.8	0.22	0.23
				R		
				y = − 0.64 x + 53.0	0.36	0.081
				nR		
				y = 2.35x − 94.2	0.91	0.001
LATsum^1^	301.1 ± 90.8	354.7 ± 65.9	0.12	All		
				y = − 0.04x + 36.6	0.22	0.21
				R		
				y = − 0.07x + 53.88	0.4	0.05
				nR		
				y = 0.26x − 94.2	0.91	0.001

IVD, Intraventricular delay; TAT, total RV-LV activaation time; LVEAT, LV electrical activation time; LAT, Local activation time; DCD, device calculated delay; ΔDCD, difference between DCD at baseline and at 6MFU.

^1^—measurement at baseline.

^2^—measurement after 6 months.

After 6 months of biventricular pacing, a negative correlation with statistical trend was found between the percentage of LVESVi reduction and the duration of stimulated QRSp complex (r = 0.32, *p* = 0.068). There was also a correlation between the percentage of LVESVi reduction and the reduction in QRS complex duration compared to baseline ΔQRS (immediately after surgery r = 0.5, *p* = 0.003) and after 6 months of follow-up (r = 0.55, *p* = 0.001). In ROC analysis, the cut-off criterion for QRSp was ≤ 120 ms (AUC = 0.785, p = 0.003), and for ΔQRS > 20 ms (AUC = 0.796, *p* < 0.001).

Initially, no differences were observed in the group of responders and non-responders in terms of the maximum delay—DCDmax base (*p* = 0.53) and the mean delay DCDmean base (*p* = 0.43). After 6 months, there was a difference in both groups with a correlation between the percentage of LVESVi reduction and the studied differences in the parameters ΔDCDmax (r = 0.51, *p* = 0.004) and ΔDCDmean (r = 0.6, *p* < 0.001). These relationships are confirmed by the C statistic results: (ΔDCDmax AUC = 0.722, *p* = 0.002), ΔDCDmean AUC = 0.881, *p* < 0.001). Regarding the intraventricular delay (IVd), a correlation was found with the percentage of LVESVi reduction after 6 months of biventricular pacing (r = 0.49, *p* = 0.003). Furthermore, a correlation was found between the percentage change in LVESVi and left ventricular electrical activation time (LVEAT). In the next stage of the analysis, this parameter showed a different trends in the responder (r = 0.49, *p* = 0.002) and non-responder groups (r = 0.71, *p* = 0.03). In the LAT analysis, baseline values did not differ between responder and non-responder groups. However, these subgroups also showed an opposite relationship to the LVESVi reduction percentage after 6 months of pacing with LATmax, LATmean, LATsum values. In the next step of the analysis, LAT was evaluated in individual segments for responders and non-responders (Table 3).

**Table 3:** Analysis of LAT values in specific segments across responders (R) and non-responders (nR).

Segment	LAT (ms)				*p*
	Responder		Non-responder		
	Median	95% CI	Median	95% CI	
Apex	19.1	8.0–26.3	14.7	0.0–32.7	*p* = 0.43
Midanterior	9.7	0.0–21.3	27.5	7.0–55.2	***p* = 0.03**
Anterobasal	29.4	36–65.5	42.9	16.0–45.0	***p* = 0.05**
Midposterior	36.9	26.7–42.5	16.3	3.6–36.7	***p* = 0.03**
Posterobasal	53.6	49.3–59.8	59.4	42.4- 78.3	*p* = 0.52
Midseptal	3.6	0.0–14.8	11.0	0.6–27.9	*p* = 0.17
Basoseptal	30.1	20.5–41.9	43.1	34.9–58.6	***p* = 0.05**
Midlateral	46.5	37.0–53.2	45.8	27.6–64.2	*p* = 0.72
Basolateral	55.9	45.1–67.7	75.4	52.1–80.5	***p* = 0.05**

Significant values are in bold.

**Figure 2:**
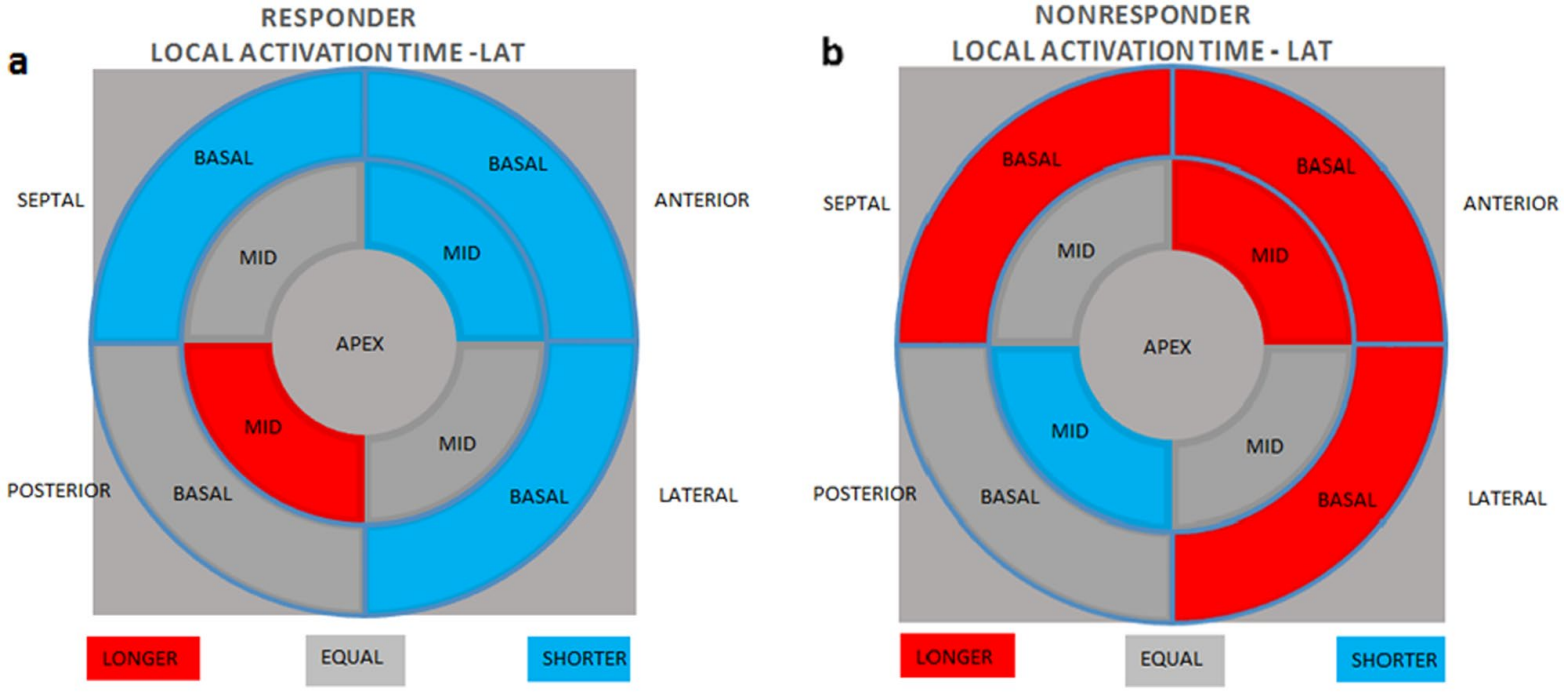
Local latency of LV segments in the responder group (**a**) and the non-responder group (**b**) in the “bull’s-eye” projection.

The LV segment excitation sequences in the “bull’s-eye” projection in the study group after analysis of segmentally averaged LAT values are shown in Fig. 2.

The described relationships are reflected in the ROC analysis, in which LAT of the posterior middle segment is a predictor of change in LVESVi percentage after 6 months of stimulation. An inverse relationship was observed in the basal areas of the lateral, anterior, and septal walls and in the middle segment of the anterior wall (Table 4).

**Table 4:** Detailed LAT analysis in LV segments.

Segment	Sensitivity (%)	Specificity (%)	Cut-off value (ms)	AUC	*p*
Anterobasal	56.5	88.9	< 32.5	0.732	0.01
Midanterior	79.2	66.7	< 24.5	0.745	0.02
Basoseptal	58.3	88.9	< 31.6	0.738	0.007
Basolateral	75.0	77.8	< 67.7	0.722	0.026
Midposterior	75.0	87.5	> 26.0	0.744	0.039

On the basis of the obtained results, prediction models were created for the individual electrocardiographic parameters presented in Table 5A and the parameters obtained with AEMM in Table 5B.

**Table 5:** Predictive model of positive response to resynchronization therapy for electrocardiographic parameters measured at baseline (A) and parameters obtained with AEMM (B).

A				B			
ROC (AUC)	0.87			ROC (AUC)	0.85		
95% CI	0.708–0.961			95% CI	0.685– 0.953		
Variable	Odds ratio	95%CI	*p*	Variable	Odds ratio	95%CI	*p*
QRSn	0.93	0.84–1.03	0.09	IVd	1.06	1.0022–1.1320	0.04
ΔQRS	1.12	1.00 -1.25	0.01	LVEAT	1.12	0.9980–1.2580	0.05
QRSp	0.99	0.93 -1.05	0.74	LATmean	0.83	0.6759–1.0122	0.06

## Discussion

Our most important finding is that certain time-related parameters taken from the ECG record, device-calculated dyssynchrony or measurements made with the AEMM, as well as specific change in selected parameters appear to be of key importance for a positive response to CRT. A favorable response to CRT can be expected in patients with baseline left bundle branch block and a QRS duration of more than 150 ms, which constitute evidence of electrical dyssynchrony^1,2^. In the study group, QRSd time was measured using standard ECG recording and NOGA HP electromechanical system mapping. A similar method was used by Carpio et al.^4^ in assessing the timing of left ventricular excitation. Because patients who met standard criteria^5^ for resynchronization system implantation were included in the study, no significant baseline differences were found between the later responder and non-responder groups for either QRSd or QRSn scores. Significant differences between responder and non-responder group appear after CRT system implantation and relate to both QRSp values and QRSd shortening—ΔQRS. The difference between baseline and stimulated QRS is more significant after 6 months in both groups and may result from favorable electrical remodeling. In the study group, a correlation was observed between the percentage of the LVESVi change and the reduction of the QRS complex duration —ΔQRS immediately after the implantation procedure as well as after 6 months of follow-up. A correlation was also observed between the percentage of LVESVi change and the QRSp complex. These results are consistent with the observation of Rickard et al. ^6^, who noted a relationship between QRS complex shortening following resynchronization pacing and its response to CRT. Similar findings were published by van Stipdonk^7^, analysing QRS complex morphology in patients with CRT system. Jastrzębski et al.^8^ describe the acute effect of QRS duration-shortening after CRT implantation as a predictor of positive response to CRT and Takaya et al.^9^ indicates the optimal cut-off point as ΔQRS = 7 ms. QRS complex duration is shortened due to improved depolarization pattern and the effect of electrical remodelling, which is a prerequisite for mechanical and structural remodeling. In our study, both QRSp and ΔQRS occurred to be predictive factors of response to CRT. The electrical impulse vector, which depends on the location of the left ventricular lead and the programmed electrical impulse configuration, is critical for the beneficial effect of resynchronization. According to the REVERSE study results, the posterior position should be preferred when implanting the left ventricular lead to achieve the most favourable haemodynamic response^10^. Similar benefits can also be expected with left ventricular lead implantation in the anterior, lateral, and posterior positions, as demonstrated in the COMPANION study^11^. All patients in our study group achieved lateral or posterolateral positioning outside the apical region, consistent with the conclusions of MADIT-CRT study^3^. Apical placement of the left ventricular lead is associated with poorer response to resynchronization pacing. According to the TARGET study^12^, pacing vector selection should be based on the largest estimated delay, and optimizing atrioventricular and interventricular delay increases the positive response rate to CRT, as emphasized by Gold et al.^13^. Stimulation based on the most delayed cathode achieves an effective form of resynchronization according to Polasek et al.^14^. Similar results were also reported by Gold et al.^2^ and Zanon et al.^15^. The PEGASUS study^16^ used an estimated interventricular delay based on the measurement algorithm of the implanted device. Acute intraoperative assessment of conduction time between left and right ventricular leads based on intracardiac recordings is also used to estimate the delay between the LV and RV^17^. It should be emphasized that the algorithms for estimating RV-LV delay do not precisely define the delay between the right and left ventricles, but only between the right ventricular lead and the individual left ventricular lead rings. This parameter shows a strong correlation with the interventricular delay measured during electromechanical mapping in the studied group of patients. The observed correlation between the percentage of LVESVi reduction after 6 months of biventricular pacing and the change in mean and maximum delay measured with device algorithm available in the Abbott Quadra Assura MP software. DCDmean and DCDmax cardioverter-defibrillators indicate favorable electrical remodeling in the responder group, and the parameters studied may be indicative of response to CRT.

The intraventricular conduction delay (IVd) described in an animal model by Strik et al.^18^ is a predictive factor of CRT response reported by many authors. Latency is most commonly measured based on a standard ECG as the time from the onset of the QRS complex to its first maximum excursion^2^, sometimes estimated by independent blinded investigators as presented by Takaya et al.^9^. Kosztin et al.^19^ proposed another way of IVd measurement using intracardiac recording while Engels et al.^20^ used mapping techniques in the assessment of intracardiac delay. In the study group, the time delay between the left and right ventricles was measured using the NOGA XP anatomical-electromechanical mapping system. The value of the IVd measured this way defined as the delay between the earliest recorded QRS excursion in V1 and the earliest left ventricular excitation recorded by the catheter within the left ventricle (LV) shows a clear relationship between the duration of QRS measured during the NOGA procedure and the delay measured by the implanted device algorithm. In the study group, the values of delay measured by NOGA XP system differed between responders and non-responders, and in logistic regression analysis, they were a predictor of response to resynchronization therapy, which was confirmed by ROC analysis with a cut-off point > 34 ms. This result is consistent with the results of Engelas et al.^20^, indicating a value above 40 ms as the cut-off point of transseptal conduction in predicting response to CRT.

The total left ventricular activation time (LVEAT) in AEMM defines the time between the earliest and latest electrical excitation of the left ventricle. There are also noninvasive methods to measure LVEAT based on electrocardiographic recordings^20^. LVEAT prolongation is proportional to baseline left ventricular electrical impulse propagation abnormalities and left ventricular damage. At the baseline, no differences in LVEAT values were observed between responders and non-responders in the study group. In the analysis after 6 months of pacing, an inverse linear relationship was observed between LVEAT and the percentage of LVESVi reduction in the responder group. Analysis of these parameters in the non-responder group showed a different, directly proportional relationship. The behaviour of this parameter reflecting the total excitation time within all LV segments, regardless of location, from the earliest recording of an electrical potential within the LV to the latest one, was explained by the analysis of the maximum, mean, and summed values of the local activation time (LAT). There is a strong correlation between LVEAT and LATmax, as well as LVEAT and LATmean and LVEAT and LATsum. LATmax reflects the time from the earliest beat to the latest segment, whereas LATmean and LATsum reflect global abnormalities in left ventricular electrical impulse transmission. LATmax values between responders and non-responders did not differ from the LVEAT analysis, whereas LATmean and LATsum showed variation in both subgroups. The lack of variation in LATmax is caused by a different location of the segments with the greatest latency, as illustrated in the “bull’s-eye” projection in Fig. 2a and b. LAT values in the apex, middle, and lateral septal segments, as well as basal posterior segments, did not differ in terms of LAT between responders and non-responders. LAT in the basal segments of the lateral, anterior, and septal walls, and in the middle segment of the anterior wall in the non-responder group is longer than in the responder group. In contrast, LAT time in the posterior wall’s middle segment is significantly longer in the responder group. The described relationships are reflected in the analysis, which draws a proportional linear relationship between the posterior middle segment’s LAT and the LVESVi percentage, changed after six months of pacing. An inverse relationship was observed in the basal areas of the lateral, anterior, and septal walls and in the anterior wall’s middle segment.

Identification of the segment with the highest LAT value and the left ventricular lead’s location in this area allows for corrections of delays and improved LV depolarization pattern. Intraoperative techniques using local activation time measured from a guidewire in potentially targeted cardiac vein with intracardiac electrocardiographic recording and the unipolar signal analysis from the guidewire presented by Rials et al.^21^ are also used to identify this area and select the target vessel. As a result, they have beneficial effects on resynchronization. An alternative method of assessing the most delayed segment is to use speckle tracking ultrasound techniques and EnsiteNavX as described by Mafi-Rad et al.^22,23^, to obtain the left ventricular lead’s optimal position LAT values of selected segments have predictive values in response to CRT.

The problem of non-response to resynchronisation therapy affecting approximately 30% of patients with an implanted CRT system remains a complex issue. Claude Bernard, a French medical research physician and physiologist who was awarded the Order of the Legion of Honour, in 1865 said words reflecting the individual patient's relationship to clinical outcomes: "The response of the 'average' patient to a therapy is not necessarily the response of the individual patient standing before the clinician". The predictive parameters of a favourable response to resynchronisation therapy allow us to identify those patients in whom we can expect an improvement after CRT implantation, but does the lack of positive results in this field fully define a non-responder and entitle us to withdraw the patient from this form of therapy? Certainly, by proper qualification based on a reliable assessment of the available broad-spectrum predictive parameters, this decision can be made, especially when we have alternative methods of cardiac resynchronisation using conduction system pacing—bundle-branch pacing (HBP) and left bundle-branch pacing (LBBP) on the one hand^24–29^ and hope for further development of new cardiac pacing techniques on the other.

## Study strengths and limitations

The value of this study lay in the use of the highly accurate NOGA XP electro-mechanical mapping system, innovative in the prediction of CRT response, and in the comparison of the obtained results with all parameters available using non-invasive methods. It should be emphasized that electro-mechanical mapping was performed only for the purpose of this study. The main limitation of the study is relatively small group of patients studied: however, due to the invasive nature of the mapping, recruitment to the study was halted when statistically significant correlations occurred.

## Summary and conclusions

The analysis of time parameters obtained by anatomo-electromechanical mapping (AEMM) can be helpful in identifying patients with a high probability of a positive response to resynchronization therapy, and the values of IVd, LVEAT and LATmean are predictive parameters of response to CRT.

The greatest delay in activation of the middle segment of the posterior wall is associated with an increased rate of positive response to resynchronization therapy. In contrast, predominant delays in the basal and middle segments of the anterior wall, as well as in the basal segments of the lateral wall and the septum, are associated with a risk of no response to resynchronization therapy.

Stimulated QRS complex time less than 120 ms and a reduction in baseline QRS complex time greater than 20 ms are predictive factors of response to resynchronization therapy. In our prediction model, the value of the reduction in the baseline QRS duration is an independent predictor of positive response to resynchronization therapy among electrocardiographic parameters.

Measurements of the delay between right and left ventricular using DCD by the CRT Toolkit Auto VectSelekt MultiVector Test algorithm allow optimal selection of the area with the greatest delay, and changes in the values of these parameters reflect favourable electrical and structural remodeling.

## Data Availability

The datasets used and/or analysed during the current study available from the corresponding author on reasonable request.

## Abbreviations

AEMM: Anatomo-electromechanical mapping
CRT: Cardiac resynchronization therapy
CRTD: Cardiac resynchronization therapy with cardioverter-defibrillator function
DCD: Device calculated delay
ΔDCD: Difference between DCD at baseline and after 6 months
ECG: Electocadiogram
ESVi: Left ventricular end systolic volume index
IVd: Intraventricular delay
HBP: His bundle pacing
LBBB: Left bundle branch block
LBBP: Left bundle branch pacing
LV: Left ventricle
QRSd: QRS duration
QRSp: Paced QRS duration
QRSn: The QRS complex time from the ECGs obtained during NOGA electromechanical mapping
ΔQRS: Difference between QRS duration at baseline and after 6 months
LVEAT: Left ventricular electrical activation time
TAT: Total activation time
LAT: Local activation time
Pt: Patient
Pts: Patients
RV: Right ventricle
6MFU: Six month follow up
